# Complete mitochondrial genome of the Xianggelila Hot-spring snake, *Thermophis shangrila* (Reptilia, Colubridae)

**DOI:** 10.1080/23802359.2016.1197072

**Published:** 2016-07-23

**Authors:** Li-Fang Peng, Shi-Yang Weng, Dian-Cheng Yang, Chang-Hu Lu, Song Huang

**Affiliations:** a Co-Innovation Center for Sustainable Forestry in Southern China, Nanjing Forestry University, Nanjing, China;; b College of Biology and the Environment, Nanjing Forestry University, Nanjing, China;; c College of Life and Environment Sciences, Huangshan University, Huangshan, China;; d School of Sciences, Tibet University, Lahsa, China

**Keywords:** Mitogenome, phylogeny, *Thremophis shangrila*

## Abstract

The Xianggelila hot-spring snake, *Thermophis shangrila* (Reptilia, Colubridae), is a species of family Colubridae, which is seen only in Shangri-La, Northern Yunnan, China. Herein, the complete mitochondrial genome of this species has been reported. The total length of the genome was 17,327 bp, and composed of 13 protein-coding genes, 22 tRNA genes, 2 rRNA genes and 2 control regions. The total base composition was 32.7% for A, 23.7% for T, 13.5% for G and 32.7% for C. The phylogenetic tree with 13 protein-coding genes of *T*. *shangrila* together with 12 other closely related species belonging to the family Colubridae was reconstructed.

Hot-spring snakes, *Thermophis*, achieve the world’s highest altitude distribution among all snakes, commonly restricted to the proximity of geothermal sites (Zhao 2006; Dorge et al. 2007; Huang et al. 2009; Hofmann 2012). This genus was erected by Malnate (1953), and only included three species (*Thermophis baileyi* Wall 1907; *Thermophis zhaoermii* Guo et al. 2008; *Thermophis shangrila* Peng et al. 2014), which was restricted to the Tibet Autonomous Region (TAR), Cogsum, Western Sichuan and Shangri-La, Northern Yunnan. In this study, we sequenced the complete mitochondrial genome sequences of *T*. *shangrila*.

The specimen of *T*. *shangila* (Voucher number: HS11192) was sampled from Shangri-La, Northern Yunnan, China. Total genomic DNA was extracted from the liver tissue of a female Xianggelila hot-spring snake using Takara MiniBEST Universal Genomic DNA Extraction Kit (Takara, Dalian, China). The genomic DNA was amplified by the polymerase chain reaction (PCR) with 13 pairs of primers described in He et al. (2010). The total length of the mitogenome (GenBank Accesson No. KU174488) was 17,327 bp, and the base composition was 32.7% for A, 23.7% for T, 13.5% for G and 32.7% for C.

The mitogenome of *T*. *shangrila* composed of 13 protein-coding genes (PCGs), 22 tRNA genes, 2 rRNA genes (12S and 16S rRNA) and 2 control regions (D-loop). Nine of the 13 PCGs used ATG as the start codon, while ND1, ND2, and ND3 used ATA codon and COX1 used GTG. The PCGs have four types of termination codon, including TAA for ND4L and ND5, AGG for COX1, ND6 and ND4, T–– for ND1, ND2, COX2, COX3, ND3 and Cytb, TA– for ATP8 and ATP6. Twenty-two tRNA genes were interspersed in the mitochondrial genome and ranged in size from 62 to 74 bp. The 12S (925 bp) and 16S rRNA (1480 bp) genes were located between tRNA*^Phe^*and tRNA *^Leu^* and separated by the tRNA*^Val^*. Two control regions found in the mitogenome of *T*. *shangrila* were 1023 and 1091 bp, respectively.

In order to convince the mitochondrial DNA sequences obtained in this study, we used the 13 PCG genes of the mitogenome of *T*. *shangrila* and other 12 closely related species, belonging to the family Colubridae to construct the phylogenetic tree. These CD sequences were concatenated as a supergene for each species. We constructed the ML tree (Figure 1) using the new supergenes in http://www.phylo.org/portal2/login!input.action. The phylogenetic analysis result was consistent with the previous research with a high support. It indicated that our first reported mitogenome sequences could meet the demand and explain some related evolution issues.

**Figure 1.**
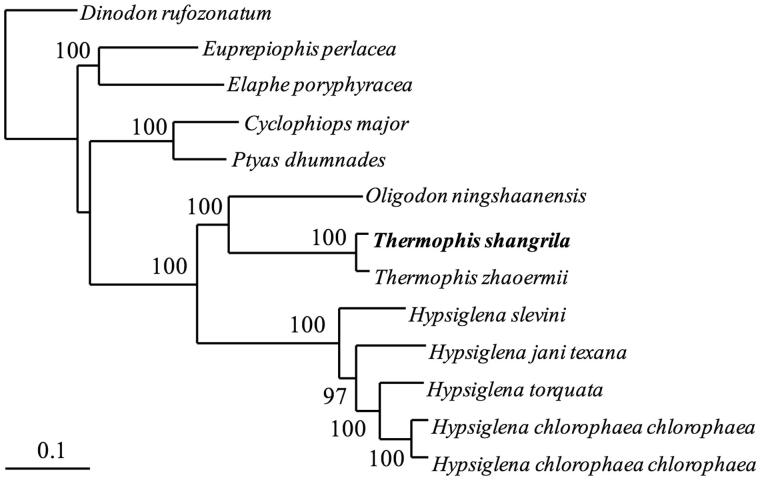
A RA × ML tree of 13 species from Colubridae was constructed based on the dataset of 13 concatenated mitochondrial PCGs. Sequence data used in the study from GenBank are the following: *Dinodon rufozonatum* (KF148622), *Euprepiophis perlacea* (KF850472), *Elaphe poryphyracea* (GQ181130), *Cyclophiops major* (KF148620), *Ptyas dhumnades* (KF148621), *Oligodon ningshaanensis* (KJ719252), *Hypsiglena jani texana* (EU728592), *Hypsiglena torquata* (EU728591), *Hypsiglena chlorophaea chlorophaea* (EU728577), *Hypsiglena slevini* (EU728584), *Thermophis zhaoermii* (GQ166168) and *Hypsiglena chlorophaea chlorophaea* (EU728593).

To date, the mitogenome shown in this study is the second species of the *Thermophis* genus whose complete mitochondrial sequence is available from GenBank, the first one was *T*. *zhaoermii* (GQ166168) (He at al. 2010). The mitogenome data in our study will help to study the evolutionary relationships and genetic diversity of *T*. *shangrila*.
